# Understanding eHealth Cognitive Behavioral Therapy Targeting Substance Use: Realist Review

**DOI:** 10.2196/20557

**Published:** 2021-01-21

**Authors:** Farhud Shams, James S H Wong, Mohammadali Nikoo, Ava Outadi, Ehsan Moazen-Zadeh, Mostafa M Kamel, Michael Jae Song, Kerry L Jang, Reinhard Michael Krausz

**Affiliations:** 1 Institute of Mental Health Department of Psychiatry University of British Columbia Vancouver, BC Canada; 2 Department of Educational and Counselling Psychology, and Special Education Faculty of Education University of British Columbia Vancouver, BC Canada; 3 Addiction Institute of Mount Sinai Department of Psychiatry Icahn School of Medicine at Mount Sinai New York City, NY United States; 4 Department of Psychiatry Tanta University Tanta Egypt; 5 Faculty of Medicine University of British Columbia Vancouver, BC Canada; 6 Department of Psychiatry University of British Columbia Vancouver, BC Canada; 7 School of Population and Public Health University of British Columbia Vancouver, BC Canada; 8 Centre for Health Evaluation and Outcome Sciences St. Paul's Hospital Vancouver, BC Canada

**Keywords:** eHealth CBT, substance use, realist review, eHealth mechanisms, opioid crisis

## Abstract

**Background:**

There is a growing body of evidence regarding eHealth interventions that target substance use disorders. Development and funding decisions in this area have been challenging, due to a lack of understanding of what parts of an intervention work in which context.

**Objective:**

We conducted a realist review of the literature on electronic cognitive behavioral therapy (eCBT) programs for substance use with the goal of answering the following realist question: “How do different eCBT interventions for substance use interact with different contexts to produce certain outcomes?”

**Methods:**

A literature search of published and gray literature on eHealth programs targeting substance use was conducted. After data extraction, in order to conduct a feasible realist review in a timely manner, the scope had to be refined further and, ultimately, only included literature focusing on eCBT programs targeting substance use. We synthesized the available evidence from the literature into Context-Mechanism-Outcome configurations (CMOcs) in order to better understand when and how programs work.

**Results:**

A total of 54 papers reporting on 24 programs were reviewed. Our final results identified eight CMOcs from five unique programs that met criteria for relevance and rigor.

**Conclusions:**

Five strategies that may be applied to future eCBT programs for substance use are discussed; these strategies may contribute to a better understanding of mechanisms and, ultimately, may help design more effective solutions in the future. Future research on eCBT programs should try to understand the mechanisms of program strategies and how they lead to outcomes in different contexts.

## Introduction

An estimated 269 million individuals worldwide used drugs in 2018, which represents a 30% increase from 2009 [1]. Globally, around 36 million individuals have a drug use disorder; however, only 1 out of 8 individuals who need substance use treatment receive it [1]. In particular, North America is currently experiencing an unprecedented epidemic of drug overdose deaths, as nearly 45,000 individuals died from an opioid overdose in the United States in 2018 and over 4000 opioid-related deaths occurred in Canada in the same year [2]. The current system of addiction care has to deal with several challenges in providing care, especially for the most vulnerable populations. One such major challenge of the system of care is the availability of resources, which is limited in respect to incidence, prevalence, and distribution of substance use conditions [3]. Many also experience barriers in accessing treatment due to discrimination and stigma, particularly those in correctional settings, ethnic minorities, immigrants, and refugees [1]. In rural areas, people do not have sufficient access to specialist care. In the Yukon, for example, assuming a psychiatrist-to-population ratio of 1:10,000 [4], there should be between 4 and 5 psychiatrists. Currently, there are only 2 full-time general psychiatrists in the territory (ie, 1.6 per 100,000 population). Nunavut and the Northwestern Territories do not have any practicing psychiatrists [5]. Given the pressing need for effective measures to tackle this public health crisis, all stakeholders are increasingly committed to developing and implementing innovative solutions.

eHealth is a major field that has attracted growing attention for its versatility and accessibility. These interventions are delivered via electronic devices, such as computers, tablets, smartphones, and other handheld devices; delivery modes include websites, email, mobile apps, text messages, and telephone calls [6]. In this review, we distinguish between web-based interventions, intended for use through an internet browser on a computer; computer-based interventions (ie, software that is run offline on a computer, for example, from a CD or DVD); and apps (ie, native iOS and Android apps). These eHealth interventions have been used to target a range of substance use problems and have demonstrated effectiveness in previous meta-analyses [7-15]. However, the development of eHealth interventions for illicit drug use is still at a more formative stage.

Potential advantages of eHealth interventions are their wide reach, particularly among subpopulations such as young people and people residing in rural areas; higher likelihood of disclosing information due to anonymity, especially given the sensitive nature of substance use; lower maintenance cost, especially for automated self-help interventions; and easy transferability of the interventions to other languages and settings [16]. Additionally, fidelity is assured if educational material is delivered via the internet, as the material is delivered in its entirety and not adapted to a teacher’s or counselor’s style of delivery. This is not an uncommon problem and may cause removal of essential components of a program. Programs may also be expanded to other settings and still delivered in a consistent manner without the need for additional resources. Despite these disadvantages, many challenges with eHealth interventions can be avoided or mitigated by identifying the optimal eHealth intervention for a certain setting and context.

Given the increasing use of eHealth interventions to tackle the overdose crisis, decision makers need to make informed decisions about future investments in eHealth for substance use. Decisions must be based on several layers of complexity. First, eHealth interventions have substantial heterogeneity in the nature of the intervention (eg, information, psychoeducation, cognitive behavioral therapy [CBT], peer based, etc), mode of delivery (eg, website vs app), level of therapist involvement (eg, automated vs therapist assisted), and price (eg, free vs paid). Second, contextual factors, such as targeted subpopulation (eg, at-risk youth, inmates, and individuals with chronic substance use disorders [SUDs]) and implementation strategy (eg, part of school curriculum, part of a rehabilitation program, and freely available online), play an important role in the outcomes of eHealth interventions. Finally, the time frame for evaluating the desired outcomes should also be determined.

Traditional evaluations and systematic reviews tend to predominantly focus on whether the programs “worked” (eg, reduced substance use), often without an understanding of the complexity of the intervention in terms of for whom they may or may not have been effective, under what circumstances, and why. The realist review methodology used in this study can answer questions with this level of complexity. In this case, the basic evaluative question “What works?” changes to “What is it about this program that works, for whom, and under what circumstances?” [17]. Mechanisms matter in this approach because they explain the process that leads to outcomes, and the context is important because it changes the processes by which an intervention produces an outcome. Therefore, both context and mechanism must be systematically examined along with the intervention’s strategy and outcome.

Realist reviews tend to start their synthesis with a wider scope and focus it during the review’s iterations. In this review, all eHealth interventions targeting substance use were explored in the first iteration, and eHealth interventions based on CBT (ie, electronic CBT [eCBT]) were chosen as the scope of the realist review in the subsequent iterations. This was due to CBT being one of the most common evidence-based interventions that can be effectively delivered in an eHealth setting, among various approaches for substance use. In order to unpack the underlying mechanisms of how specific intervention strategies work in specific contexts, an important first step in a realist review is to narrow down, clarify, and refine the scope and, thus, the review question [17]. The aim of this realist review was to answer the following question: “How do different eCBT interventions for substance use interact with different contexts to produce certain outcomes?”

## Methods

### Realist Methodology

The methodology for this review has been adapted from Pawson et al [17], with an additional round of scoping to refine and clarify the research question in order to be able to conduct a feasible realist synthesis. Our research question stems from the following fundamental realist question: “What works for whom and why?” Specifically, “In an environment in which certain *contextual factors (C)* are at play, program activities or *strategies (S)* are implemented. These strategies, in combination with the contextual factors, trigger certain internal processes or changes in the participants’ way of thinking, which is the *mechanism (M)* that, in turn, triggers the desired *outcome (O)*” [18]. Identifying program strategies and the mechanisms they trigger, alongside the context in which they are implemented, will allow us to generate knowledge that is useful for the implementation of programs and research that regulate outcomes in specific contexts. One intervention has multiple program strategies and each can trigger one or more mechanisms in different contexts that cause desirable or undesirable outcomes [17].

In this review, we also made use of the reconceptualization proposed by Dalkin et al [19], who argue that realist syntheses have difficulty deciding which aspects of an intervention contribute contextually or mechanistically to a Context-Mechanism-Outcome configuration (CMOc). It is important for both aspects to come together to be able to fully explain a program from a realist perspective.

**Figure 1:**
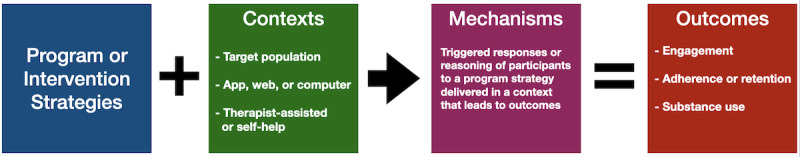
Overview of results according to the Context-Mechanism-Outcome formula proposed by Dalkin et al [19].

By adding program strategy (ie, mechanism resource) as a variable between context and mechanism response (see Figure 1), we can be more accurate in how we construct our CMOcs and ensure that the mechanism is described more completely in terms of which resource triggers which response [19].

### Search

We conducted a search on two electronic medical databases—MEDLINE and Embase—on June 26, 2018. The results were limited by published year (ie, 2009 to present) and by language (ie, English). The three main reasons for initially limiting the scope of the literature search in this study were as follows: (1) the tendency of realist reviews to develop a creeping nature consuming excessive amounts of time and resources, (2) the time-sensitive nature of the review given the relevance of its results to the opioid crisis, and (3) limited available resources. Moreover, we restricted the literature search to the past 10 years to ensure inclusion of current and relevant eHealth programs. The search strategy consisted of three baskets of key terms: substance use– and addiction-related keywords, eHealth- and web-related keywords, and the three keywords prevention, intervention, and treatment. Keywords under each group were combined with the “OR” operator, while the groups were merged with the “AND” operator. The three baskets helped in limiting results to find papers that focused on interventions for substance use using web-, computer-, or mobile-based components. The search returned 3184 records (see Multimedia Appendix 1). A gray literature search using Google and the search string “web-based substance intervention” was limited to the first 500 results and was completed on July 3, 2018.

### Screening

Two reviewers independently screened the titles and abstracts of all database results. Disagreements were discussed between the reviewers and if not resolved, a third party in the research team was consulted. Inclusion criteria were as follows: (1) the paper examined web- or computer-based interventions, (2) the intervention targeted substance use (ie, alcohol, tobacco, cannabis, opioids, and stimulants), (3) the intervention tool was not being used for an electronic medical record or an electronic health record, (4) the intervention was not delivered via a wearable device (eg, watch or pedometer), and (5) if the intervention had telephone calls or text messaging as part of the intervention, it was also accompanied by a web-based component. Interventions that targeted only mental health conditions without a substance use component were not included. After removal of duplicates and screening, 186 records were included: 161 from MEDLINE and Embase and 25 from the Google search. References of key papers were tracked, and experts were consulted to identify additional relevant articles that were missed by the search. An additional 55 articles were included through reference tracking and expert consultation, resulting in a total of 241 articles being reviewed in full.

### Quality Appraisal and Data Extraction

Data extraction began with the use of an appraisal form to ensure the fit of the papers for this review by examining rigor and relevance (see Multimedia Appendix 2). The form was adapted from the National Collaborating Centre for Methods and Tools appraisal form, in addition to consultation with the research team by pretesting the form to ensure its utility. Initially, the team also appraised two articles together and discussed the results as a team to ensure a consistent approach for this process. In realist reviews, the appraisal process is not based on the standardized evaluation of methodology that is typically used in systematic reviews (ie, randomized controlled trial studies possess the highest methodological standard) [17]. Rather, the appraisal process is based on the reviewer’s judgement of the fitness of an article for that specific synthesis [17]. The assessment of methodological soundness and trustworthiness of the paper is based on the appropriateness of the methodology with what was stated as the goal or objective of the study. This rigor was assessed using the following questions: (1) Did the authors have clearly defined research goals? (2) Were the research goals adequately addressed in the results and discussion sections of the article? (3) Was the methodology used in the study appropriate with respect to the research goals? and (4) Overall, did the study have reliability and validity? Following these criteria, a study may be excluded for reasons such as the sample size being too small or too homogeneous (eg, only female). Relevance was ensured by considering whether the paper had a direct relevance to our research project by contributing to forming the program theory. During this process, 18 articles were excluded because they did not meet rigor and/or relevance requirements. Furthermore, after excluding study protocols and those articles that were not accessible, 208 articles met the initial review inclusion criteria and were used for data extraction.

Data extraction was completed with a data extraction template that collected information on intervention details, study setting and context, results, and mechanisms. During this process, it became clear that the scope of the synthesis was too broad, given the limited time and resources available. To address that, articles that were of the *review* type (ie, systematic reviews), protocols, and cost-effectiveness studies were excluded from the synthesis.

After preliminary data extraction, we further narrowed down the scope to only focus on CBT-based programs. First, all studies that looked at eHealth interventions that were based on a CBT theoretical framework were identified. Second, studies were excluded if (1) they solely addressed alcohol and/or tobacco use with no other substance use, (2) they were review papers, and (3) they did not identify or discuss any mechanisms of change. Alcohol and tobacco use eHealth interventions have a large body of literature devoted to them and they tend to target a wide array of populations, which may result in very heterogeneous contexts compared to other substance use interventions. This study sought to narrow down its focus to illicit substances, therefore excluding nicotine and alcohol. It is important to note that recreational cannabis use was still illegal in Canada at the time of the review and is still illegal in most countries around the world, including in the United States under federal law; therefore, these studies were included in this review. A total of 54 articles met the eligibility criteria after refining the scope and were included in the final synthesis.

### Context-Mechanism-Outcome Configurations

In order to study how different strategies and contexts produce particular outcomes, each reviewer extracted candidate CMOcs based on qualitative discussions found in the identified literature that described how an intervention or parts of an intervention may or may not work. The context and outcomes of the paper were summarized in order to create full CMOcs that included a program strategy, context, mechanism, and outcome. After identifying all candidate CMOcs, along with their program strategies, the quality of each CMOc was appraised using the following criteria: (1) How rigorous and specific was the CMOc? (2) Was it possible to make a clear distinction between program strategies (ie, mechanism resource) and mechanism response? and (3) How relevant was the CMOc in improving the understanding of, and providing strategies to improve, eCBT interventions for substance use? Reviewers analyzed studies for themes in the candidate CMOcs using keywords identified in the mechanisms, outcomes, and program strategies. Four reviewers rated and discussed the quality of the candidate CMOcs (ie, rigor and relevance) and, in consultation with the principal investigator, candidate CMOcs were reduced to a final set.

## Results

A total of 54 papers from 24 unique programs that are grounded in CBT theory were reviewed. Eight CMOcs from five unique programs met the inclusion criteria and were included in the final synthesis.

Table 1 summarizes the eight CMOcs that were included from the reviewed literature regarding eCBT interventions [20-31] . The study setting, which includes how and where the study was carried out, is an important part of the intervention context. For example, the Computer-Based Training for Cognitive Behavioral Therapy (CBT4CBT) studies were delivered as a blended model combining CBT4CBT with treatment as usual, such as methadone maintenance treatment for opioid use disorder, as well as having a research associate available at all times to answer questions related to the program. The Self-Help for Alcohol and Other Drug Use and Depression (SHADE) and CBT for insomnia (CBT-I) interventions, on the other hand, were delivered to participants with the assistance of therapists or clinicians. Out of the five programs, two were delivered in a web-based format, two were computer-based interventions (eg, using CDs or DVDs in an offline context), and one was delivered as an app. Most interventions were targeting individuals with SUDs.

**Table 1:** Summary of components of the eight Context-Mechanism-Outcome configurations (CMOcs).

CMOc No.	Context of intervention	Program strategy	Mechanism	Outcome	Program
1	Individuals with substance use disorder (SUD) No human involvement; self-help electronic cognitive behavioral therapy (eCBT)	The program focused on strengthening coping skills	Improvement in coping skills, strengthening of executive cognitive control, and reduction of attentional bias toward drug-related cues	Reduced cue-induced craving, resulting from a reduction of attentional bias Retention in treatment Drug use and abstinence	CBT4CBT^a^ [20-23]
2	Individuals with SUD and possible cognitive impairment No human involvement; self-help eCBT	The program material was prepared for higher literacy levels and normal levels of cognitive functioning	Negative effect on self-efficacy and commitment to abstinence, specifically in people with cognitive impairment	Reduced retention in treatment Higher dropout rates	CBT4CBT [24,25]
3	Individuals with SUD accessing Breaking free Online (BFO) drug and alcohol treatment services across the United Kingdom No human involvement; self-help eCBT	The program provided tailored feedback on the level of impairment on each module	Recognition of problem areas	Increased use of modules that were highlighted red (ie, highest level of impairment)	BFO [26]
4	Individuals with SUD accessing BFO drug and alcohol treatment services across the United Kingdom No human involvement; self-help eCBT	Completion of a higher *dose* of modules was facilitated by the program	*Dosage effect* (ie, more intervention tasks completed will lead to improved outcomes)	Increased improvement in functioning	BFO [26]
5	Individuals with SUD accessing BFO drug and alcohol treatment services across the United Kingdom No human involvement; self-help eCBT	Participants were given cognitive restructuring training	Recognition of dysfunctional beliefs	Reduced severity of alcohol dependence	BFO [26]
6	Individuals with current comorbid depression and problematic alcohol and cannabis use Therapist clinician–assisted eCBT	The program was designed as a self-help intervention	Feelings of empowerment and possible enhancement in problem-solving skills	Increased client initiative and acceptability	SHADE^b^ [27,28]
7	Veterans with cannabis use disorder and current sleep problems Therapist clinician–assisted eCBT	Participants were reminded to track behavior	Feelings of accountability	Increased adherence to tracking behavior	CBT-I^c^ Coach [29,30]
8	Veterans No human involvement; self-help eCBT	Users participated in focus groups and individual feedback sessions to inform development of the program	Relatability and relevance of content to target population (ie, veterans)	Improved likability, ease of use, and relevance of the program Increased engagement	CHMF^d^ [31]

^a^CBT4CBT: Computer-Based Training for Cognitive Behavioral Therapy.

^b^SHADE: Self-Help for Alcohol and Other Drug Use and Depression.

^c^CBT-I: cognitive behavioral therapy for insomnia.

^d^CHMF: Coming Home and Moving Forward.

CBT4CBT is a computer-based program to help people stop or reduce the use of substances, including alcohol. The two studies, from four papers, included in this synthesis had 77 and 101 participants with mean ages of 40.6 and 43.1 years, respectively. A total of 40% and 60% of participants were female, respectively. We found that CBT4CBT, provided as part of a blended model—treatment as usual (eg, methadone treatment + CBT4CBT)—of substance use treatment in the clinic for people with SUD, can improve retention in treatment and abstinence by strengthening participants’ coping skills and executive cognitive control, as well as reducing attentional bias (ie, CMOc 1). The authors associate a reduction in attentional bias with a reduction in cue-induced craving [20-23].

Additionally, for CBT4CBT, two additional studies were included. Participants in the two studies had a mean age of 42.3 and 38.3 years, respectively. Sample sizes were 52 and 120, of which 40% and 33.3% were female, respectively [23,24]. We found that cognitive impairment had a negative influence on retention in treatment through affecting participant change mechanisms, such as self-efficacy and commitment to abstinence (ie, CMOc 2). Preparing material for low literacy levels can possibly address this issue to some extent [32].

Breaking Free Online (BFO) is a web-based relapse-prevention program that targets individuals with SUD. One major study with a large sample (N=2311) was included in our synthesis and resulted in three CMOcs. The mean age of the participants in this study was 42.2 years (range 15-76) and 45% of the participants were female. We found that providing tailored symptom feedback on biopsychosocial impairment (see Lifestyle Balance Model in Davies et al [33]) that highlights areas the user needs to work on will increase usage of those modules by the user, because users are able to recognize the importance of working on those areas with significant levels of impairment (ie, CMOc 3) [26].

In the BFO study, we also found that the greater number of modules completed, the greater the improvement reported in functioning, due to the so-called *dosage effect* (ie, CMOc 4). The dosage effect indicates that an increase in the number of tasks or intervention strategies completed will lead to improved outcomes; in this case, a greater improvement in biopsychosocial functioning [26].

Finally, in the BFO study, we found that in an eCBT intervention, cognitive restructuring can help lower the severity of alcohol dependence because it targets “dysfunctional beliefs that underpin and maintain unhelpful and unhealthful behaviours” (ie, CMOc 5) [26].

The SHADE intervention is a computer-delivered motivational treatment for individuals with co-occurring SUD and depression. The mean age of the participants in the SHADE study was 35.37 years (range 18-61), and 54% of the participants were female in a total sample of 97. In the therapist clinician–delivered CBT group, 19 out of 35 (54%) participants completed all sessions. In the computer-delivered group, 15 out of 32 (47%) participants completed all sessions. A total of 30 participants were not assigned to any treatment group. The self-help nature of the program, which allows users to take charge of their own intervention, increased client initiative and, thus, treatment acceptability through a sense of empowerment and enhanced problem-solving skills (ie, CMOc 6). Client initiative is one of the subcategories of treatment acceptability and describes the extent to which a client feels that they are directing therapy. The authors suggest that higher levels of client initiative are associated with changes in alcohol use. It has also been suggested that the self-help nature of a treatment leads to more sustainable outcomes in the longer term [27,28].

CBT-I Coach is a mobile app designed to be an adjunct to in-person CBT-I. Two studies of CBT-I Coach were included. The first study had 4 male veterans with a mean age of 47 years (SD 16.31, range 27-65). The veterans diagnosed with DSM-5 (Diagnostic and Statistical Manual of Mental Disorders, Fifth Edition) cannabis use disorder and sleep problems were randomized to receive a 2-week intervention with (1) the CBT-I Coach mobile app (n=2) or (2) a placebo, control mood-tracking app (n=2). In the second study, the participants’ mean age was 48.50 years (SD 14.93). The total sample size was 18 and 39% of the participants were female. The CBT-I Coach studies suggest that including regular reminders (eg, daily notifications) can help increase adherence to the tracked behavior, because individuals feel like they are being held accountable to the program (ie, CMOc 7) [29,30].

Coming Home and Moving Forward (CHMF) is a web-based self-help program for recent combat veterans with posttraumatic stress disorder and substance use. Mean age and range were not reported, but veterans were either part of focus groups (n=18) or individual feedback sessions (n=34). Each group had 4 female participants. The veterans that participated in this study found a web-based intervention based on CBT principles to be likable, easy to use, and relevant to their experiences, because focus group feedback helped to make the intervention more contextually anchored in their experiences (CMOc 8) [31].

## Discussion

### Principal Findings

In this review, we sought to answer the following question based on the original realist review question: “How do different eCBT interventions for substance use interact with different contexts to produce certain outcomes?” The review revealed some strategies that could be used in some contexts in order to improve substance use outcomes. The results of the review identified five strategies that may be considered when developing or implementing an eCBT intervention targeting substance use.

The first strategy is to consider addressing cognitive functioning and/or impairment. There is a body of evidence on the potential impact of cognitive functioning on treatment response and substance use outcomes in web- or computer-based CBT interventions [34-37]. Some authors suggest that more than 50% of people entering substance use treatment have some level of impairment [37-39]. Assessing neuropsychological functioning and tailoring content to the appropriate level of cognitive functioning is, therefore, important in order to improve understanding of the material. Simplifying content or targeting impairment directly via cognitive training are two other strategies [40,41]. The program strategy identified in CMOc 3 may be applied to this kind of impairment as well.

The second strategy is to tailor content to the user’s needs. Tailored content may help improve engagement and other treatment outcomes [42]. One strategy identified in a program that addressed cognitive functioning (ie, CMOc 3) assessed levels of impairment in the aspects of the Lifestyle Balance Model [33] and provided participants with tailored feedback, highlighting the most important modules to work on. This resulted in highlighted modules being worked on more, and those modules resulted in greater improved treatment outcomes in comparison to modules that were worked on less [26].

Third, cognitive restructuring has been effectively used in dealing with negative thoughts [43]. There is evidence that the delivery of cognitive restructuring material can be effective in a digital context. In a study conducted on BFO [26], completion of the *negative thoughts*, cognitive restructuring, Lifestyle Balance Model intervention strategies was associated with improved outcomes across all measures, including severity of alcohol dependence, depression, anxiety, and quality of life.

Fourth, our review revealed that despite increasing focus on including users’ experiences and perspectives in the development of new solutions, existing solutions were more often adaptations and digitalization of conventional treatment approaches. This does not leave much room for involvement of users in forming the digital solution. One program targeting veterans used focus groups to make the intervention more tailored to the veterans’ needs. As a result, veterans described the program as likable, easy to use, and relevant to their experiences [31]. This shows the importance of including the target population in the early stages of the intervention design.

Finally, engagement is a key ingredient that needs attention in order to increase usage, retention, and adherence to an eHealth intervention [42,44]. This review identified multiple strategies that can be used to improve engagement of users with the program. The *dosage effect* explains that an increase in usage of modules will lead to greater improvement in desired outcomes [26]. Facilitating the completion of intervention strategies and modules should be a key focus of eCBT interventions. Additionally, if utilized well, the self-help nature of some interventions may be an advantage, as it may increase client initiative and, thus, acceptability of the program (ie, CMOc 6). Making sure additional criteria for acceptability are met, such as ease of use and an intuitive system design (eg, by applying cocreation principles in the design and development of interventions), can further help to improve engagement and other treatment outcomes. As described in CMOc 7, timely reminders can also play an important role in improving engagement with programs.

### Limitations

The majority of publications in this field reported on randomized controlled trials. An issue with conducting a realist review on these types of publications is that their primary focus is on quantitative results without any attempt to interpret the mechanisms. Thus, any realist review must generally focus on screening of the discussion section of publications to identify author opinions or any qualitative information that may shine a light on the mechanisms of how certain interventions work. Very few of the studies published in the literature on eHealth CBT interventions for substance use provided enough information to investigate the theory and the mechanisms that drove the intervention outcomes in different contexts. As such, most of the information was inferred from an appraisal of the authors’ comments, and subjectivity of these inferences must be acknowledged. Only one study statistically analyzed mechanisms of action [26].

Another limitation is the lack of reporting of more proximal outcomes in the studies; for example, retention or adherence outcomes, changes in perception, willingness to change, and self-efficacy. Although long-term goals, such as reducing substance use, are crucial for understanding effectiveness of interventions, proximal outcomes may be of help when trying to understand the mechanisms of action behind the effects.

### Conclusions

To our knowledge, this is the first published realist review that aims to identify what works for whom in eCBT interventions that target SUD. The review identified eight CMOcs from five programs, and we discussed five strategies that we have identified through synthesis of the findings from the CMOcs: addressing cognitive functioning, tailoring content to user needs, addressing negative thoughts, cocreation, and addressing engagement. These strategies, among others, may be considered when developing or making funding decisions regarding eCBT programs for SUD. eCBT interventions are an increasingly popular tool for program developers for addressing substance use problems, yet additional research is needed to identify what factors lead to desired outcomes and to explain the mechanisms of change. Thus, future studies should focus on clearly defining the components of an intervention, recording these mechanisms, and defining their relationship to outcomes and context, in order to better understand what components of a program work and why.

## Appendix

Multimedia Appendix 1 Initial literature review search strategy.

Multimedia Appendix 2 Appraisal form.

## Abbreviations

BFO: Breaking Free Online
CBT: cognitive behavioral therapy
CBT4CBT: Computer-Based Training for Cognitive Behavioral Therapy
CBT-I: cognitive behavioral therapy for insomnia
CHMF: Coming Home and Moving Forward
CMOc: Context-Mechanism-Outcome configuration
DSM-5: Diagnostic and Statistical Manual of Mental Disorders, Fifth Edition
eCBT: electronic cognitive behavioral therapy
SHADE: Self-Help for Alcohol and Other Drug Use and Depression
SUD: substance use disorder
