# Supplementary material for: Understanding eHealth Cognitive Behavioral Therapy Targeting Substance Use: Realist Review
Source: J Med Internet Res. 2021 Jan 21;23(1):e20557. doi: 10.2196/20557 (PMC7861997; doi:10.2196/20557)
Supplement: Multimedia Appendix 1 [file jmir_v23i1e20557_app1.docx]

## Initial literature review search strategy

## Ovid EMBASE

| 1 | substance use*.mp. |
| --- | --- |
| 2 | substance addict*.mp. |
| 3 | substance depend*.mp. |
| 4 | substance abus*.mp. |
| 5 | substance misuse*.mp. |
| 6 | drug use*.mp. |
| 7 | drug abuse*.mp. |
| 8 | drug misuse*.mp. |
| 9 | drug addict*.mp. |
| 10 | drug depend*.mp. |
| 11 | addiction/ |
| 12 | "substance use"/ |
| 13 | substance abuse/ |
| 14 | "drug use"/ |
| 15 | drug abuse/ |
| 16 | drug dependence/ |
| 17 | drug misuse/ |
| 18 | illicit drug/ |
| 19 | opioid use*.mp. |
| 20 | opioid depend*.mp. |
| 21 | opioid abuse*.mp. |
| 22 | opioid misuse*.mp. |
| 23 | opioid addict*.mp. |
| 24 | opiate use*.mp. |
| 25 | opiate depend*.mp. |
| 26 | opiate abuse*.mp. |
| 27 | opiate misuse*.mp. |
| 28 | opiate addict*.mp. |
| 29 | heroin use*.mp. |
| 30 | heroin depend*.mp. |
| 31 | heroin abuse*.mp. |
| 32 | heroin misuse*.mp. |
| 33 | heroin addict*.mp. |
| 34 | opiate/ |
| 35 | opiate addiction/ |
| 36 | opiate dependence/ |
| 37 | heroin dependence/ |
| 38 | online*.mp. |
| 39 | telemedicine*.mp. |
| 40 | internet*.mp. |
| 41 | web*.mp. |
| 42 | smartphone*.mp. |
| 43 | e-health*.mp. |
| 44 | e-mental health*.mp. |
| 45 | emental health*.mp. |
| 46 | computer*.mp. |
| 47 | mobile*.mp. |
| 48 | app.mp. |
| 49 | Internet/ |
| 50 | web browser/ |
| 51 | computer program/ |
| 52 | smartphone/ |
| 53 | mobile application/ |
| 54 | intervention.mp. |
| 55 | prevention.mp. |
| 56 | treatment.mp. |
| 57 | early intervention/ |
| 58 | prevention/ |
| 59 | 1 or 2 or 3 or 4 or 5 or 6 or 7 or 8 or 9 or 10 or 11 or 12 or 13 or 14 or 15 or 16 or 17 or 18 |
| 60 | 19 or 20 or 21 or 22 or 23 or 24 or 25 or 26 or 27 or 28 or 29 or 30 or 31 or 32 or 33 or 34 or 35 or 36 or 37 |
| 61 | 38 or 39 or 40 or 41 or 42 or 43 or 44 or 45 or 46 or 47 or 48 or 49 or 50 or 51 or 52 or 53 |
| 62 | 54 or 55 or 56 or 57 or 58 |
| 63 | 59 and 60 and 61 and 62 |

## Ovid Medline

| 1 | substance use*.mp. |
| --- | --- |
| 2 | substance addict*.mp. |
| 3 | substance depend*.mp. |
| 4 | substance abus*.mp. |
| 5 | substance misuse*.mp. |
| 6 | drug use*.mp. |
| 7 | drug abuse*.mp. |
| 8 | drug misuse*.mp. |
| 9 | drug addict*.mp. |
| 10 | drug depend*.mp. |
| 11 | addiction/ |
| 12 | "substance use"/ |
| 13 | substance abuse/ |
| 14 | "drug use"/ |
| 15 | drug abuse/ |
| 16 | drug dependence/ |
| 17 | drug misuse/ |
| 18 | illicit drug/ |
| 19 | opioid use*.mp. |
| 20 | opioid depend*.mp. |
| 21 | opioid abuse*.mp. |
| 22 | opioid misuse*.mp. |
| 23 | opioid addict*.mp. |
| 24 | opiate use*.mp. |
| 25 | opiate depend*.mp. |
| 26 | opiate abuse*.mp. |
| 27 | opiate misuse*.mp. |
| 28 | opiate addict*.mp. |
| 29 | heroin use*.mp. |
| 30 | heroin depend*.mp. |
| 31 | heroin abuse*.mp. |
| 32 | heroin misuse*.mp. |
| 33 | heroin addict*.mp. |
| 34 | opiate/ |
| 35 | opiate addiction/ |
| 36 | opiate dependence/ |
| 37 | heroin dependence/ |
| 38 | online*.mp. |
| 39 | telemedicine*.mp. |
| 40 | internet*.mp. |
| 41 | web*.mp. |
| 42 | smartphone*.mp. |
| 43 | e-health*.mp. |
| 44 | e-mental health*.mp. |
| 45 | emental health*.mp. |
| 46 | computer*.mp. |
| 47 | mobile*.mp. |
| 48 | app.mp. |
| 49 | Internet/ |
| 50 | web browser/ |
| 51 | computer program/ |
| 52 | smartphone/ |
| 53 | mobile application/ |
| 54 | intervention.mp. |
| 55 | prevention.mp. |
| 56 | treatment.mp. |
| 57 | EARLY MEDICAL INTERVENTION/ |
| 58 | "EARLY INTERVENTION (EDUCATION)"/ |
| 59 | 1 or 2 or 3 or 4 or 5 or 6 or 7 or 8 or 9 or 10 or 11 or 12 or 13 or 14 or 15 or 16 or 17 or 18 |
| 60 | 19 or 20 or 21 or 22 or 23 or 24 or 25 or 26 or 27 or 28 or 29 or 30 or 31 or 32 or 33 or 34 or 35 or 36 or 37 |
| 61 | 38 or 39 or 40 or 41 or 42 or 43 or 44 or 45 or 46 or 47 or 48 or 49 or 50 or 51 or 52 or 53 |
| 62 | 54 or 55 or 56 or 57 or 58 |
| 63 | 59 and 60 and 61 and 62 |
