# Supplementary material for: Understanding eHealth Cognitive Behavioral Therapy Targeting Substance Use: Realist Review
Source: J Med Internet Res. 2021 Jan 21;23(1):e20557. doi: 10.2196/20557 (PMC7861997; doi:10.2196/20557)
Supplement: Multimedia Appendix 2 [file jmir_v23i1e20557_app2.doc]

**First Author**: __________________________

**Second Author**: ________________________

**Year**: _________

**Journal**: ______________________________

**Reviewer**: ____________________________

**Realist Review Appraisal Form**

**Instructions:** Please read the selected article and come back to this form to answer the questions below.

**Rigour**

1. Do you think, the author(s) had a clearly focused question/issue/goal?

☐ Yes ☐ No

How confident are you in your answer (1= Not all confident, 5= Very confident): _______

Please provide a brief explanation, if you answered “No”:

_________________________________________________________________________________________________________________________________________________________________________________________________________________________________________________________________________________________________________________________________________________________________________________________________________________________________________

1. Was this question/issue/goal addressed in the article?

☐ Yes ☐ No

How confident are you in your answer (1= Not all confident, 5= Very confident): _______

Please provide a brief explanation, if you answered “No”:

_________________________________________________________________________________________________________________________________________________________________________________________________________________________________________________________________________________________________________________________________________________________________________________________________________________________________________

1. Do you think, the methodology used in the study was appropriate with the question/issue/goal?

☐ Yes ☐ No ☐ Not Applicable

How confident are you in your answer (1= Not all confident, 5= Very confident): _______

Please provide a brief explanation, if you answered “No”:

_________________________________________________________________________________________________________________________________________________________________________________________________________________________________________________________________________________________________________________________________________________________________________________________________________________________________________

1. Do you think, the study overall is reliable and has validity?

☐ Yes ☐ No ☐ Not Applicable

How confident are you in your answer (1= Not all confident, 5= Very confident): _______

Please provide a brief explanation, if you answered “No”:

_________________________________________________________________________________________________________________________________________________________________________________________________________________________________________________________________________________________________________________________________________________________________________________________________________________________________________

**Relevance**

Do you think, the selected paper has relevance to our research project?

☐ Yes ☐ No

How confident are you in your answer (1= Not all confident, 5= Very confident): _______

Please provide a brief explanation for your answer:

_________________________________________________________________________________________________________________________________________________________________________________________________________________________________________________________________________________________________________________________________________________________________________________________________________________________________________
